# SSTR2 as an anatomical imaging marker and a safety switch to monitor and manage CAR T cell toxicity

**DOI:** 10.1038/s41598-022-25224-z

**Published:** 2022-12-03

**Authors:** Yago Alcaina, Yanping Yang, Yogindra Vedvyas, Jaclyn E. McCloskey, Moonsoo M. Jin

**Affiliations:** 1 Molecular Imaging Innovations Institute, Department of Radiology, Weill Cornell Medicine, BB-1500, 413 E. 69th St., New York, NY 10065 USA; 2 Department of Surgery, Weill Cornell Medicine, New York, NY 10065 USA

**Keywords:** Imaging the immune system, Cancer immunotherapy

## Abstract

The ability to image adoptively transferred T cells in the body and to eliminate them to avoid toxicity will be vital for chimeric antigen receptor (CAR) T cell therapy, particularly against solid tumors with higher risk of off-tumor toxicity. Previously, we have demonstrated the utility of somatostatin receptor 2 (SSTR2) for CAR T cell imaging, illustrating the expansion and contraction of CAR T cells in tumor as well as off-tumor expansion. Using intercellular adhesion molecule 1 (ICAM-1)-specific CAR T cells that secrete interleukin (IL)-12 as a model, herein we examined the potential of SSTR2 as a safety switch when combined with the SSTR2-specific maytansine-octreotate conjugate PEN-221. Constitutive secretion of IL-12 led to continuous expansion of CAR T cells after rapid elimination of tumors, causing systemic toxicity in mice with intact MHC expression. Treatment with PEN-221 rapidly reduced the abundance of CAR T cells, decreasing the severity of xenogeneic graft-versus-host disease (GvHD), and prolonged survival. Our study supports the development of SSTR2 as a single genetic marker for CAR T cells that is readily applicable to humans both for anatomical detection of T cell distribution and an image-guided safety switch for rapid elimination of CAR T cells.

## Introduction

Autologous T cells modified to express chimeric antigen receptor (CAR) have shown a remarkable treatment efficacy against hematological cancers. So far four different CD19-specific CAR T cells have been approved against indications of diffuse large B-cell lymphoma (DLBCL), B-cell acute lymphoblastic leukemia (B-ALL), mantle cell lymphoma (MCL), and follicular lymphoma^1–4^. More recently, two CAR T cells specific to B-cell maturation antigen (BCMA) have been approved to treat multiple myeloma^5,6^. In comparison, the advances of CAR T cells against solid cancers have been less remarkable. The main challenges faced by CAR T cell therapy include the immunosuppressive features of the tumor microenvironment, antigen escape of the cancer cells, and the scarcity of tumor-specific antigens^7^. In addition, CAR T cell therapy could trigger toxicities, affecting both hematological and solid tumor cancers. These toxic effects range from mild to lethal, and include cytokine release syndrome (CRS), neurologic toxicity, and on-target off-tumor toxicity^8^.

Several approaches have been tested to avoid organ or systemic toxicity caused by CAR T cells. Some specifically address CRS and neurotoxicity, like the blockades of cytokines (GM-CSF, IL-1, IL-6) or catecholamines^9–11^. Others, like dasatinib administration, affect all T cells of the organism by disrupting the signaling cascade downstream of CD3ζ and ZAP70^12^.

For selective targeting of the CAR T cells, various engineered “off-switch” genes have been investigated. When the cells express one of these genes, an external molecule is introduced into patients to act as an antidote against the CAR T cells and therefore alleviate the provoked toxicities. Monoclonal antibodies (mAb) could be used against engineered epitopes, for example cetuximab against truncated epidermal growth factor receptor (tEGFR) or rituximab against CD20^13,14^. Another approach is the expression of the enzymes by the CAR T cells that can convert innocuous compounds into toxic ones and therefore succumb to cell death. For instance, cells carrying herpes simplex virus type 1 thymidine kinase (HSV1-tk) that metabolize the nucleoside analog ganciclovir are killed by DNA damage after treatment with ganciclovir^15^. Similarly, cytosine deaminase allows CAR T cells to convert 5-fluorocytosine into highly toxic 5-fluorouracil, eliciting cell death^16^. An alternative approach is the introduction of inducible apoptotic genes, such as caspase-9 and Fas, that can induce cell death on the CAR T cells when expressed in the presence of a dimerizing agent such as rimiducid^17,18^.

To accurately use the aforementioned strategies we require information about the CAR T cell distribution and activities related to associated toxicities. Systemic toxicity such as CRS caused by CAR T cells against hematological cancers can be readily predicted by detection of CAR T cells in circulation or blood markers such as IL-6, C-reactive protein, or IFN-*γ*^19^. However, in the setting of solid tumors, anatomical detection of CAR T cell location will be critical as the spatiotemporal expansion of CAR T cells cannot be fully captured by traditional blood pharmacokinectic analyses. Several genetic markers, such as HSV1-tk^20^, the human sodium-iodide symporter (hNIS)^21,22^, the prostate-specific membrane antigen (PSMA)^23,24^, and *Escherichia coli* dihydrofolate reductase enzyme (eDHFR)^25^, have been explored for T cell imaging and potentially as a suicide switch. Among them, HSV1-tk has been translated to clinical use, wherein ^18^F-FHBG ([fluoro-3-(hydroxymethyl) butyl]guanine) for positron emission tomography (PET) imaging and ganciclovir for CAR T cell elimination^20,26–28^. However, the viral nature of HSV1-tk raises potential immunogenicity risks, and the HSV1-tk-specific radiotracers have substantial normal tissue uptake^29^.

In this study, we propose somatostatin receptor 2 (SSTR2) as a dual genetic marker for imaging and elimination of CAR T cells. SSTR2 has physiologic expression restricted to neuroendocrine tissues, and is found to be overexpressed in some tumors including neuroendocrine, carcinoid, and small cell lung cancers^30,31^. This expression pattern has provided an opportunity for imaging and killing these tumors by targeting SSTR2 with ^68^Gallium (^68^Ga)- and ^177^Lutetium (^177^Lu)-conjugated octreotide peptide, respectively^32–34^. Previously, we designed a single lentiviral vector for expression of CAR and SSTR2, and demonstrated the utility of SSTR2 as a genetic marker for anatomical detection of CAR T cell distribution in vivo^35,36^. Our trackable CAR T cell therapy targeting intercellular adhesion molecule 1 (ICAM-1) is currently being evaluated in a Phase 1 trial against advanced thyroid cancer (ATC) for safety and the feasibility of CAR T cell imaging using ^68^Ga-DOTATATE (ClinicalTrials.gov Identifier: NCT04420754). In this study, to demonstrate the utility of SSTR2 as a suicide switch, we additionally engineered CAR T cells to constitutively secrete interleukin (IL)-12 to stimulate the growth and function of T cells, potentially increasing both the antitumoral effect and the related toxicities^37^. The elevated proliferation and toxic effects of the CAR T cells in the ATC model provided a rigorous platform to test our ability to track in vivo and to eliminate these cells using SSTR2 as a suicide gene. To seek alternative to ^177^Lu radiotherapy (^177^Lu-DOTATATE, Lutathera, Novartis), we examined a SSTR2-specific drug conjugate (the maytansine-octreotate conjugate PEN-221, Tarveda^38,39^), which is currently being evaluated for safety and efficacy against SSTR2-overexpressing advanced gastroenteropancreatic, lung, thymus, or other neuroendocrine tumors and against small cell lung cancer or large cell neuroendocrine carcinoma of the lung (ClinicalTrials.gov Identifier: NCT02936323).

## Results

### PEN-221 induces dose-dependent SSTR2 internalization and specific cytotoxicity against SSTR2-expressing Jurkat T cells

**Figure 1:**
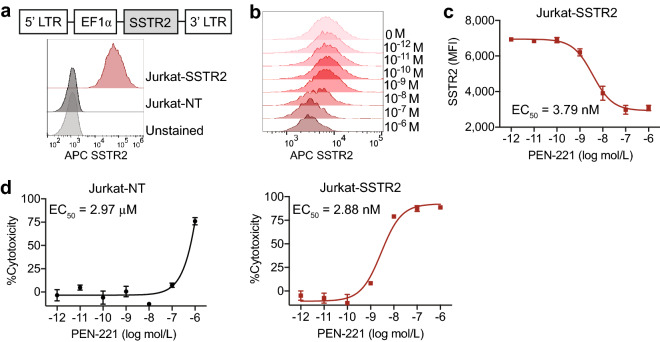
PEN-221 induces internalization of SSTR2 and exhibits specific cytotoxicity against SSTR2-expressing Jurkat cells in vitro. (**a**) Schematic representation of the lentiviral vector encoding human SSTR2 (SSTR2). Histograms show the expression of SSTR2 in non-transduced (NT) Jurkat and Jurkat-SSTR2 cells determined by flow cytometry. (**b**) Representative flow-cytometric measurements of SSTR2 internalization in Jurkat-SSTR2 cells after treatment with 10-folds serially diluted PEN-221 (10^–6^ to 10^–12^ M). (**c**) EC_50_ curve of SSTR2 internalization. The mean fluorescence intensity of surface SSTR2 is indicative of the internalization of the receptor. (**d**) Cytotoxicity of PEN-221 against SSTR2-expressing Jurkat T cells, using Jurkat-NT cells as control. Data were presented as the mean ± SD of triplicate wells.

SSTR2 belongs to the G-protein coupled receptor superfamily, and exhibits rapid internalization after binding its ligand, somatostatin^40^. To examine the activity of PEN-221 against SSTR2-expressing T cells, we transduced Jurkat T cells with a lentiviral vector to overexpress human SSTR2 receptor (Fig. 1a). Consistent with our previous observations of SSTR2 internalization induced by its agonists octreotide and lanreotide^35,41^, PEN-221 stimulated dose-dependent internalization of SSTR2 receptor in Jurkat T cells, proving that octreotide-maytansine conjugate PEN-221 preserved the function of SSTR2 agonist (Fig. 1b,c). The internalization of PEN-221 receptor complex led to dose-dependent cellular cytotoxicity and inhibition of cell proliferation. SSTR2-expressing Jurkat T cells showed high sensitivity to PEN-221, with a nanomolar EC_50_ (2.88 nM). Non-transduced Jurkat T cells that had no SSTR2 expression were much less susceptible to killing by PEN-221 with an EC_50_ > 1 μM, indicating antigen specificity of the cytotoxic effect (Fig. 1d). These in vitro results demonstrated that PEN-221 triggers SSTR2 internalization and exerts cytotoxic effect that is dependent upon SSTR2 binding.

**Figure 2:**
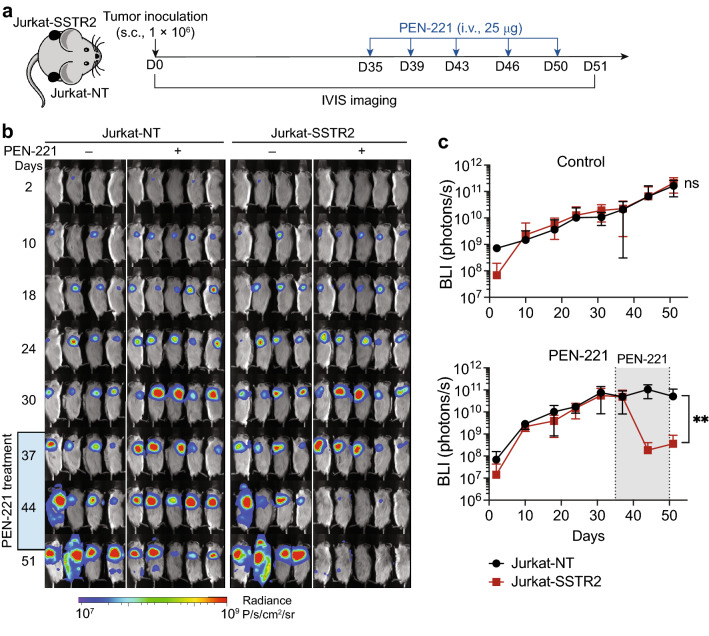
PEN-221 treatment mediates SSTR2-dependent elimination of Jurkat xenografts in vivo. (**a**) Schematic of the bilateral Jurkat mouse model. Fluc-expressing Jurkat-NT and Jurkat-SSTR2 cells (1 × 10^6^/xenograft) were implanted subcutaneously into opposite flanks of the same NSG-MHC-KO mice and treated with 25 μg of PEN-221 intravenously every 3–4 days from day 35 to day 50 or left untreated. (**b**) Tumor growth was assessed by bioluminescence imaging on a weekly basis. (**c**) Tumor burden was estimated by the level of total body bioluminescence intensity (*n* = 4 for control, and 5 for PEN-221 cohort). Data represent mean ± SD. Statistical significance was determined by unpaired, two-tailed student’s t-test. **, P < 0.01; ns, not significant.

To further evaluate SSTR2-specific cytotoxicity of PEN-221 in vivo, we used a bilateral tumor model by subcutaneously implanting Jurkat-SSTR2 and Jurkat-NT cells into the opposite flanks of the same NSG-MHC-KO mice (Fig. 2a). Without PEN-221 treatment, Jurkat-SSTR2 and Jurkat-NT tumors showed progressive and comparable tumor growth, indicating that the expression of SSTR2 has no impact on cell growth (Fig. 2b,c). As anticipated, mice treated with PEN-221 after three doses exhibited nearly complete regression of the Jurkat-SSTR2 tumor. However, PEN-221 also stunted the growth of Jurkat tumors even without SSTR2 expression, which was particularly more apparent after five cycles of injections (Fig. 2b,c). The growth inhibition of SSTR2-negative Jurkat tumors should be due to non-specific uptake of PEN-221, similar to what was seen with the killing of Jurkat-NT cells in vitro at higher concentrations of PEN-221 (Fig. 1d). Overall, more drastic elimination of Jurkat-SSTR2 tumors in vivo and a 1,000-fold higher potency of PEN-221 against SSTR2-positive Jurkat cells were consistent with prior studies that examined the specificity of PEN-221 against SSTR2^39^.

### PEN-221 treatment mitigates CAR T-associated toxicity in vivo and extends survival

**Figure 3:**
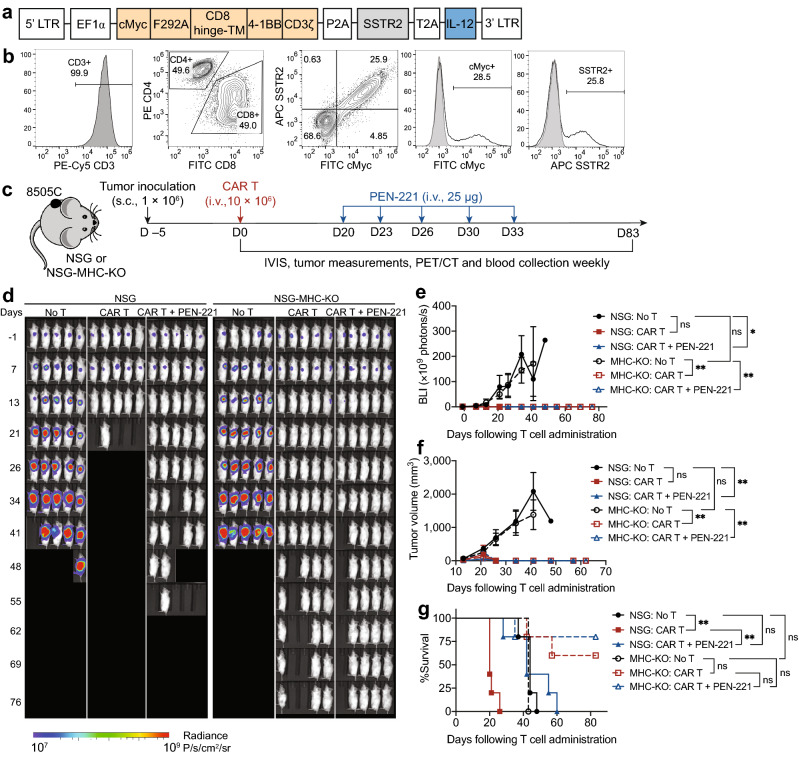
PEN-221 treatment mitigates CAR T-associated toxicity in vivo and elongates survival. (**a**) Schematic of the tricistronic lentiviral vector encoding the 2nd generation ICAM1-specific CAR (F292A-4-1BB-CD3ζ), human SSTR2 and human IL-12. Transgenes are separated by P2A and T2A ribosomal skipping sequences. F292A, a micromolar affinity I domain derived from LFA-1; CD8 hinge-TM, CD8 hinge and transmembrane domains; 4-1BB, 4-1BB costimulatory domain; CD3ζ, CD3ζ signaling domain. (**b**) Flow cytometry data showing CD3, CD4/CD8 subsets as well as CAR and SSTR2 expression detected by anti-cMyc and anti-SSTR2 antibodies. (**c**) Schematic of the ATC mouse model. Fluc-expressing 8505C cells (1 × 10^6^/mouse) were implanted subcutaneously into the upper left flank of NSG and NSG-MHC-KO mice and treated 5 days later with 10 × 10^6^ primary CAR T cells or left untreated (No T). When mice started to show symptoms of toxicity, 25 μg PEN-221 was intravenously administered every 3–4 days from day 20 to day 33. (**d**) Tumor growth was assessed by bioluminescence imaging on a weekly basis. (**e**) Tumor burden was estimated by the level of total body bioluminescence intensity. (**f**) Tumor volume was measured and plotted. Data represent mean ± SD of 5 mice per cohort. A two-way ANOVA with Tukey’s multiple comparisons test was used for statistical analysis. *, *P* < 0.05; **, *P* < 0.01; ns, not significant. (**g**) Kaplan–Meier survival curves. Statistical significance was determined by Log-rank (Mantel-Cox) test. *, *P* < 0.05; **, *P* < 0.01; ns, not significant.

In order to assess the cytotoxic effect of PEN-221 against CAR T cells in vivo and its potential to reduce the related toxicities, we established a mouse ATC model and used the 2nd generation ICAM1-specific micromolar affinity CAR (F292A-4-1BB-3ζ^36^) T cells that co-express human SSTR2 and human IL-12 (Fig. 3a,b). ATC subcutaneous tumors were generated by the inoculation of Fluc-expressing 8505C cells into the upper left flank of NSG and NSG-MHC-KO mice (Fig. 3c)^42^. The immunocompromised NSG mice and the mouse strain with additional MHC class I and class II deficiency (NSG-MHC-KO) provide models useful to distinguish between xenogeneic GvHD and on-target off-tumor toxicity. GvHD occurs when the T cell receptor (TCR) from grafted T cells (human CAR T cells in our model) recognize antigens presented by MHC on the host’s healthy tissues. Since these NSG-MHC-KO mice are resistant to GvHD, only the more clinically relevant CAR-mediated on-target off-tumor toxicities would be observed in this model.

CAR T cells manufactured with healthy donor leukopak cells were intravenously injected five days after the tumor implantation. CAR T cell-mediated tumor control was seen by bioluminescence imaging 1 week later, with almost complete tumor remission after 2 weeks in both mouse strains (Fig. 3d–f). In order to mimic the emergency clinical scenario where toxicity emerges rapidly, we delayed PEN-221 treatment until 20 days after CAR T cell infusion when severe toxicity began to occur, characterized by ruffled fur and lethargy. All 5 CAR T-treated NSG mice without PEN-221 required euthanasia within 25 days after CAR T cell administration, while all NSG-MHC-KO mice were alive and healthy. In comparison, tumor-bearing NSG mice without T cell treatment (No T) exhibited progressive growth of tumors, yet survived beyond 40 days until reaching the humane endpoint (e.g., > 2000 mm^3^ tumor volume). Therefore, we reasoned that the earlier death of CAR T-treated NSG mice without PEN-221, at a time point (~ day 20) when tumors had been completely eliminated, was not tumor-related but largely due to CAR T-associated toxicity. The NSG mice that were treated with PEN-221 rapidly recovered from severe GvHD toxicity, and the majority (4 out of 5) survived during PEN-221 treatment. Although the infusion product contained a smaller fraction of T cells expressing CAR (~ 28%, Fig. 3b), the rapid alleviation of GvHD by elimination of only CAR T cells by PEN-221 alludes to CAR T cells being the major cause for GvHD. CAR T cells might have undergone rapid expansion due to antigen stimulation and secretion of IL-12. However, mice began showing GvHD symptoms soon after the termination of PEN-221 treatment, and eventually all required euthanasia (Fig. 3d,g). This indicated that five cycles of PEN-221 treatment over 2 weeks was still insufficient to completely eliminate CAR T cells. In contrast to severe GvHD in NSG mice, GvHD associated symptoms were totally absent in NSG-MHC-KO mice and no survival differences were observed between CAR T cell cohorts with or without PEN-221 treatment (Fig. 3g).

### PEN-221 treatment eliminates CAR T cells in vivo

**Figure 4:**
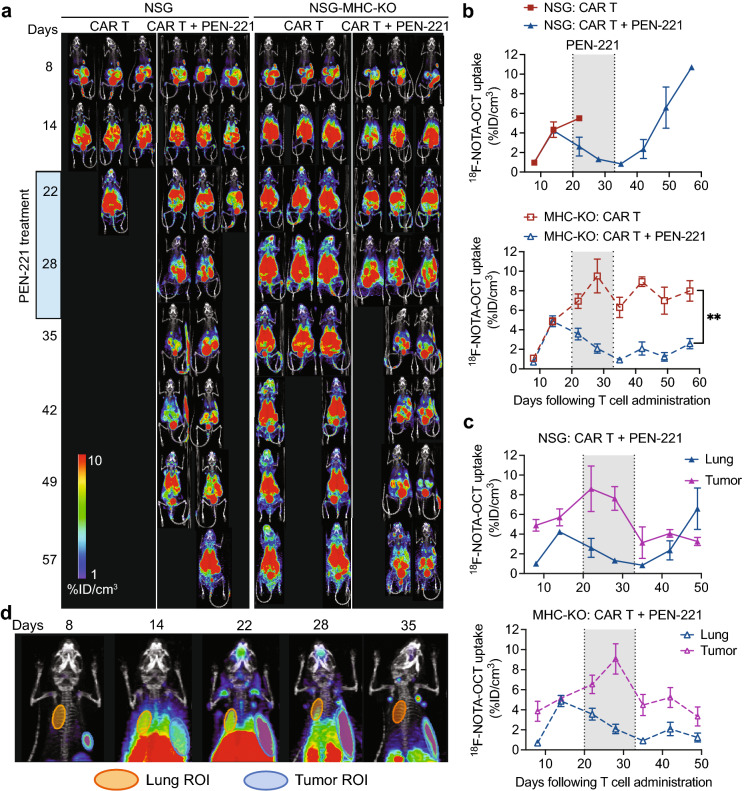
PET/CT imaging using ^18^F-NOTAOCT reveals rapid clearance of CAR T cells by PEN-221. (**a**) PET/CT imaging showing CAR T cell expansion and contraction. Images are maximum intensity projections of the entire mouse body. (**b**) Systemic expansion of CAR T cells was estimated by ^18^F-NOTAOCT update in the lungs. Data represent mean ± SD of 3 mice per cohort. A two-way ANOVA with Tukey’s multiple comparisons test was used for statistical analysis. **, *P* < 0.01. (**c**) Comparison of CAR T cell expansion and elimination in the lungs and tumors. (**d**) Representative magnified PET images (mouse# 3 in NSG-MHC-KO: CAR T + PEN-221 cohort). Region of interests (ROIs) for the lungs and tumors are shown as yellow and blue ellipses.

Distribution and expansion of CAR T cells in vivo were monitored weekly by PET/CT imaging (Fig. 4). Rapid localization of CAR T cells at tumor sites was observed 8 days after CAR T cell infusion, which was followed by systemic expansion by 14 days after CAR T cell infusion. PET/CT imaging performed 2 days after PEN-221 (day 22) clearly indicated rapid yet incomplete elimination of CAR T cells in lungs, liver, and lymph nodes. After four more cycles of PEN-221, further reduction of CAR T cells was apparent (day 35) and the majority of CAR T cells expanded in lungs and liver were eliminated. In comparison, in mice without PEN-221 CAR T cells continued to expand in the major organs (lungs, liver, spleen) and in circulation (Fig. 4a,b). We also noted significant discrepancy in the rate of CAR T elimination in lungs compared to that in site of tumor growth. In contrast to significant reduction of CAR T cell abundance in the lungs after a single administration of PEN-221 (day 22), CAR T cell density at the tumor site continued to increase before eventually subsiding after five cycles of PEN-221 (day 35) (Fig. 4c,d). In NSG mice, CAR T cells bounced back after termination of PEN-221 treatment, leading to relapse of GvHD symptoms. By contrast, CAR T cell density in NSG-MHC-KO strain remained low after the withdrawal of the drug, in both tumors and lungs (Fig. 4). These results indicate that CAR T cell recurrence seen in NSG strain was to a great extent due to TCR-MHC driven T cell stimulation. No correlation between the presence of CAR T cells and GvHD associated symptoms or survival was found on NSG-MHC-KO mice.

### Human cytokines in serum reflect expansion and killing of CAR T and tumor cells

**Figure 5:**
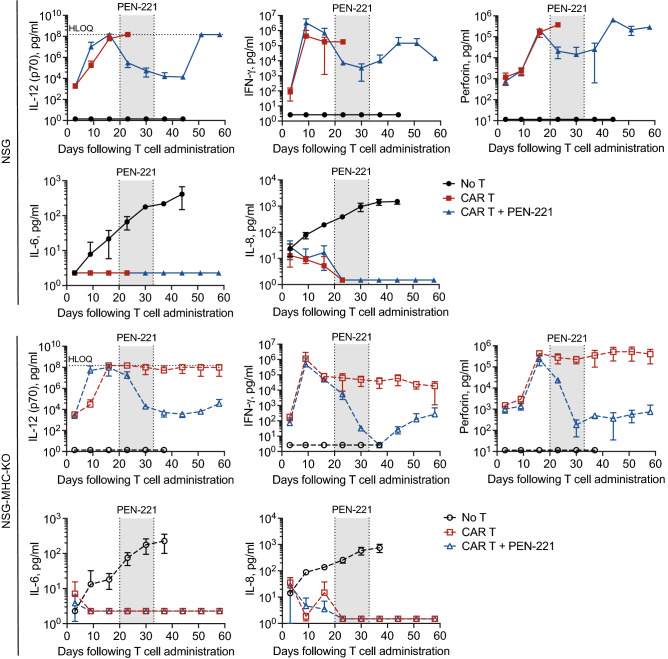
Serum cytokine analysis corroborates the prompt elimination of CAR T cells by PEN-221. Serum cytokines were measured on a weekly basis for 60 days post CAR T cell infusion. Data represent mean ± SD of 3 mice per cohort. No T, no CAR T cell treatment. HLOQ, higher limit of quantification.

The kinetics of serum cytokines closely corroborates the CAR T cell dynamics observed by PET/CT (Fig. 5). IL-12, IFN-*γ* and perforin levels in blood increased during the first week after CAR T cell infusion. These levels dropped markedly during PEN-221 treatment but remained high in untreated mice. After PEN-221 withdrawal, IFN-*γ* and perforin levels rose rapidly back to pre-PEN-221 levels in NSG mice, while IL-12 more gradually increased back to pre-PEN-221 levels. In comparison, the levels of IL-12, IFN-*γ* and perforin in NSG-MHC-KO strain slowly rose after PEN-221 withdrawal and stayed at significantly lower levels. Serum IL-6 and IL-8 were found to closely reflect tumor growth and killing, with observation of logarithmic increase in No T mice as tumor progressed. Consistent with complete tumor elimination by 2-weeks of CAR T cell infusion with no sign of relapse, IL-6 and IL-8 dropped to below 2 pg/ml in CAR T cohorts with or without PEN-221 treatment. We indeed measured high levels of IL-6 (1.17 ± 0.16 ng/ml) and IL-8 (2.98 ± 0.63 ng/ml) in the supernatant of 24 h 8505C culture (5 × 10^3^ cells in 200 μl), supporting them as indicators of tumor progression and remission (Supplementary Fig. 1). Serum levels of IL-2 and TNF-α were below 1 pg/ml in all cohorts.

## Discussion

In this study we demonstrated the feasibility of SSTR2 as a genetic marker that couples a safety switch for CAR T cell ablation with an imaging reporter for PET. CAR T cell treatments have become a promising therapy for hematological cancers with six different treatments already approved by the FDA^1–5^. Safety is still one of the biggest concerns for the translation to clinic due to CAR T cell related toxicities, like life-threatening CRS or on-target off-tumor toxicities^8^. In recent years, numerous approaches have been taken to develop CAR T cell therapies that avoid these adverse events while keeping their therapeutic efficacy^43^. Among them, the inclusion of suicide switches that allow specific ablation of CAR T cells in case of undesirable effects could be crucial for an efficient and safe application of this therapy in the clinic.

We chose SSTR2 as a suicide switch based on its limited expression in healthy tissues^30,31^, the availability of a specific cytotoxic drug conjugate^33,38^, and our previous results using it as an imaging marker for detection of CAR T cell distribution in vivo^35^. In addition, two radiotracers (^68^Ga-DOTATATE, ^64^Cu-DOTATATE) have been approved for human use, and other alternatives (e.g., ^68^Ga-DOTATOC, ^18^F-NOTA-Octreotide) that are widely available provide comparable imaging sensitivity and specificity. SSTR2 could potentially be used as a suicide switch using the FDA-approved radiotherapy (^177^Lu-DOTATATE^44^) or investigational SSTR2-specific drug conjugate PEN-221^38^. Due to the differential radiosensitivity of T cells versus tumors, the use of ^177^Lu-DOTATATE to eliminate unwanted CAR T cells will require a dose–response study to find the therapeutic window to eliminate CAR T cells while minimizing nephrotoxicity^45,46^. In comparison, PEN-221 is a conjugate consisting of maytansine, a potent microtubule-targeted cytotoxic agent, conjugated with the somatostatin analog Tyr3-octreotate. PEN-221 delivers its cytotoxic activity through internalization triggered by specific binding to surface SSTR2 in cells and binding to tubulin^39^. PEN-221 has proved effective when targeting tumor cells and is being evaluated for clinical safety and efficacy against cancers that express SSTR2^39,47^.

In our current work, we demonstrated the ability to use PEN-221 to eliminate SSTR2-expressing CAR T cells both in vitro and in vivo. In vitro, Jurkat cells transduced to express SSTR2 showed high sensitivity to PEN-221 treatment at nanomolar concentration. In vivo, biweekly intravenous injections of PEN-221 were enough to eliminate much of CAR T cells and their activity, as confirmed by both PET imaging and cytokine analysis. Efficient elimination of systemically expanded CAR T cells by PEN-221 led to drastic elongation of survival of NSG mice, a timing of PEN-221 intervention chosen after the onset of systemic toxicity. However, five cycles of PEN-221 treatment over 2-weeks were insufficient to completely eliminate CAR T cells, evidenced by recovery of CAR T cells in both NSG and NSG-MHC-KO strains. The magnitude and the rate of rebound were significantly greater in NSG strain, indicating that the expansion of CAR T cells was profoundly driven by the activation signals between TCR and MHC. Furthermore, we observed that while systemically expanded CAR T cells outside of the tumor regions were immediately reduced even after a single administration of PEN-221, whereas tumor-infiltrating CAR T cells were more resistant to PEN-221, appeared contracting only after 2–3 rounds of PEN-221. The reason for the discrepancy of T cell susceptibility to PEN-221 at off-tumor versus tumor site is unclear, and could be simply due to the difference of PEN-221 distribution, which may be more limited within and in the vicinity of the tumor sites with less developed blood vessels. Alternatively, CAR T cells at tumor sites, even after tumor was totally eliminated, might have still retained the activation or proliferation signals that counteract the cytotoxic effect of PEN-221. More immediate elimination of CAR T cells at off-tumor site and well perfused major organs may be advantageous in a situation to suppress potential toxicity while retaining the activity of tumor-infiltrating CAR T cells.

ICAM-1 is overexpressed in a range of malignant cancers, but is also expressed at lower levels in healthy cells like endothelial cells, immune cells, and some epithelial cells^48^. Our ICAM-1 CAR was built and affinity-tuned using the I domain derived from lymphocyte function-associated antigen 1 (LFA-1) and demonstrated to possess identical affinity toward human and mouse ICAM-1^41^. Despite the cross-reactivity of CAR T cells against ICAM-1, we have not observed any significant off-tumor expansion of CAR T cells in our previous xenograft models including systemic ATC 8505C and gastric cancer Hs 746T^36,42,49^. Even in NSG with normal MHC expression, the occurrence of xenogeneic GvHD was relatively rare, occurring only after ~ 2 months after CAR T infusion^49^. In the same study, however, we observed that the incorporation of inducible IL-12 into ICAM-1 targeting CAR T cells injected intraperitoneally invariably caused GvHD occurring ~ 1 month after delivery^49^. Apparently, the onset and severity of xenogeneic GvHD appear to depend on several factors including the dose and timing of human T cell infusion, the mode of injection, and the degree of T cell expansion. For example, severe allogeneic GvHD was caused by MHC minor-mismatched CD19 CAR T cells only when leukemia was present in the recipient mice, wherein CAR-target interactions induced T cell expansion^50^***.*** To validate the dual function of SSTR2 as an imaging reporter and a suicide switch, we have used subcutaneous tumors to clearly delineate tumor-infiltrating CAR T cells by PET/CT from those expanding outside of tumor. CAR T cells intravenously delivered against subcutaneous tumors, however, rarely caused GvHD in recipient mice (e.g., EpCAM-specific CAR against subcutaneous gastric cancer^51^). We have found that the constitutive secretion of IL-12 in conjunction of CAR led to robust elimination of hard-to-kill subcutaneous tumors, and at the same time caused continued expansion of CAR T cells outside of tumors. Under the influence of systemically expanding CAR T cells, different immunocompromised mouse strains showed very diverse toxicity effects. While NSG mice suffered clear symptoms of GvHD, pronounced as ruffled fur and lethargy, NSG-MHC-KO mice survived despite comparable expansion of CAR T cells. The stark difference in toxicity between the two strains with or without MHC expression provided evidence that the severe toxicity observed in NSG mouse model was largely due to xenogeneic reaction, *i.e.*, human TCR recognition of mouse peptide/MHC. This is consistent with the prior study that used genome-edited CAR T cells deficient in expression of TCR, which prevented the incidence of xenogeneic GvHD^52^. The lack of toxicity in NSG-MHC-KO strain in our study further supported our previous observation that affinity-tuned CAR T cells against ICAM1 neither reacted with ICAM1 expressed in mouse tissues despite its cross-reaction between human and murine ICAM1^41,53^ nor elicited cytokine release syndrome (CRS). However, we acknowledge that the NSG mouse models (with defective macrophages) used in this study likely preclude CAR T cell toxicities driven by cytokines and other cell types such as macrophages that are responsible for IL-6 secretion and CRS. Therefore, a mouse model that preserves functional macrophages and the use of CAR T to mimic clinical CRS should provide a more stringent condition to test the utility of our image-guided suicide switch^50^.

The main advantage of our system is the ability to use the same marker for both an imaging probe and a suicide switch. Without a reliable imaging method, we can only depend on indirect measures of the CAR T cell presence, such as cytokine evaluation or analysis of circulating T cells. These methods are inadequate for solid tumors and do not give us spatiotemporal information of the CAR T cell distribution. Being able to evaluate the proliferation and biodistribution of the CAR T cells in vivo would give us the opportunity to address the potential toxicity problems before the appearance of symptoms, as well as to monitor the response to treatment. This would allow us to optimize the CAR T cell modulation or ablation, developing personalized treatments to avoid toxicities without compromising the antitumoral effect. However, CAR T cell response to PEN-221 treatment may differ from the tumors naturally overexpressing SSTR2. Furthermore, a clinical use of PEN-221 to eliminate CAR T cells may differ from the dose–response determined from the ongoing trials against SSTR2 positive cancer patients (NCT02936323). Finding an optimal dose of PEN-221 to be used as a safety switch for CAR T cells will significantly be aided by spatiotemporal mapping of CAR T cells in patients by PET/CT. Recently, HSV1-tk has been proposed as a dual marker for CAR T cell imaging and elimination^28^. In this system ganciclovir can be used as an elimination agent, killing the cells that express the enzyme by DNA damage. The dependence on interfering DNA/RNA synthesis and consequently slower cell death could be a downside. Also, the viral origin of HSV-TK could be a major obstacle due to the immunogenicity reported in human studies^29^. On the other hand, FHBG, the imaging probe used for PET-based imaging, is not approved for human use nor as is widely available as SSTR2-specific radiotracers.

Overall, our study presents proof-of-principle evidence for the use of SSTR2 as a dual marker for CAR T cell imaging and elimination. Both the imaging probe specific to SSTR2 and the elimination agent, PEN-221, have been clinically approved or being tested, which makes this method ready to translate to the clinic. Our system offers a widely applicable strategy for treatment of different cancers that could lead to more efficient and safer CAR T cell therapies.

## Materials and methods

### Cell lines

The human thyroid cancer cell line 8505C was obtained from Sigma in 2018; HEK 293T and Jurkat T cell lines were purchased from the American Type Culture Collection (ATCC) in 2019. 8505C and Jurkat T cell lines were cultured in RPMI-1640 (Corning) supplemented with 10% heat-inactivated fetal bovine serum (FBS); HEK 293T cell line was cultured in Dulbecco's Modified Eagle's Medium (DMEM, Corning) supplemented with 10% FBS. 8505C and Jurkat T cell lines were stably transduced with a Firefly Luciferase-F2A-GFP (FLuc-GFP) lentivirus (Biosettia) for in vivo assessment of tumor burden in mouse models. All cell lines were maintained at 37 °C in a 5% CO_2_ atmosphere and were routinely validated to be Mycoplasma free using a MycoAlert™ detection kit (Lonza).

### Vector construction

The CAR construct contained from the 5′-LTR end: a c-Myc tag, a micromolar affinity inserted (I) domain (F292A) derived from lymphocyte function-associated antigen (LFA1) for ICAM1 targeting, a CD8 hinge and transmembrane domain, a 4-1BB costimulatory domain and a CD3ζ signaling domain. The genetic marker SSTR2 and stimulatory cytokine IL-12 were introduced at the C-terminus of the CAR using a porcine teschovirus-1 2A (P2A) and a Thosea asigna virus 2A (T2A) ribosome-skipping sequence to obtain comparable expression of CAR, SSTR2 and IL-12. This tricistronic construct was then cloned into a third-generation lentiviral vector backbone (VectorBuilder Inc., Vector Design Studio) under the control of EF1α promotor. A construct containing only SSTR2 was also generated and cloned into the same lentiviral backbone.

### Recombinant lentivirus production and T cell transduction

Lentivirus was produced by transient transfection of HEK 293T cells with LV-MAX™ lentiviral packaging mix (Thermo Fisher) and corresponding transfer plasmid using Lipofectamine™ 3000 transfection reagent (Thermo Fisher). The media were replaced with Opti-MEM I Reduced Serum Media (Gibco) 6 h post-transfection. The lentiviral supernatant was harvested 48 h post-transfection and concentrated with Amicon ultra centrifugal filter units (Millipore). Jurkat T cells were transduced by resuspending 1 × 10^5^ cells in 200 μl medium at a multiplicity of infection (MOI) of 3. The culture medium was refreshed after 24 h incubation, and the cells were incubated for an additional 48 h before use. Primary T cell transduction was performed as described previously^51^. Briefly, CD4^+^ and CD8^+^ T cells were enriched from human leukopaks of healthy donors (Biological Specialty Corporation) and activated with human T-activator CD3/CD28 Dynabeads (Gibco) at a 1:1 bead to cell ratio and cultured in complete T cell growth medium (TexMACS medium (Miltenyi Biotec) supplemented with 5% human AB serum (Sigma), 12.5 ng/mL IL-7 (Miltenyi Biotec), and 12.5 ng/mL IL-15 (Miltenyi Biotec)). T cells were transduced twice with lentivirus at 24 and 48 h after activation at MOI of 6, followed by expansion in 50 ml bioreactor tubes (TubeSpin, TPP) at 1−3 × 10^6^ cells/ml on a tube roller (Thermo Fisher) with a setting of 5 rpm. T cell products were harvested when 100-folds T cell expansion was achieved, typically on day 9 or 10, and cryopreserved in a 1:2 mixture of T cell complete growth medium and CS10 (STEMCELL) for in vitro and in vivo experiments.

### Flow cytometric analysis

To assess cell-surface expression of CAR and SSTR2, transduced cells were stained with FITC anti-c-myc antibody (Miltenyi Biotec, clone SH1-26E7.1.3) and APC anti-human SSTR2 antibody (R&D systems, clone 402038). T lymphocytes and sub-populations were identified using a cocktail of anti-human PE-Cy5 CD3, anti-human PE CD4 and anti-human FITC CD8 antibodies (Biolegend, clone UCHT1; RPA-T4; RPA-T8). Live cells were selected using calcein blue (Sigma-Aldrich) staining along with forward- and side-scatter gating. Flow cytometry was performed on a Gallios flow cytometer (Beckman Coulter, Inc.) and analyzed with FlowJo software (Tree Star, Inc.).

### In vitro cytotoxicity and internalization assay

The SSTR2-expressing Jurkat T cells and non-transduced Jurkat T cells (NT) were plated in 96 well at a density of 1 × 10^5^ cells/well and treated with 10-folds serially diluted PEN-221 (a kind gift from Tarveda Therapeutics) starting at a dose of 1 µM. After 4 h PEN-221 treatment, cells were washed three times and resuspended in fresh medium. The plate was further incubated at 37 °C in a 5% CO_2_ atmosphere up to 72 h. After 72 h of incubation, cells were analyzed by flow cytometry after staining with APC anti-human SSTR2 antibody (R&D systems, clone 402038) and calcein blue (Sigma-Aldrich). Calcein blue negative cells were determined to be dead target cells. The percentage of cytotoxicity was calculated using the equation: %Cytotoxicity = 100 × (experimental death – spontaneous death)/(100 – spontaneous death), where spontaneous death represents cell death from the control group (0.5% DMSO). To evaluate SSTR2 internalization induced by PEN-221, the mean fluorescence intensity (MFI) of SSTR2 was determined for each treatment condition. EC_50_ curves for cytotoxicity and SSTR2 internalization were generated using the nonlinear regression analysis with GraphPad Prism 9. Each experiment was performed in triplicate.

### In vivo mouse studies

Male mice between 5 to 7-week-old were purchased from the Jackson Laboratory and housed in the Animal Core Facility at Weill Cornell Medicine. Two different strains were used: NOD-scid IL2Rg^null^ (NSG, stock no. 005557) and NSG-B2M^null^ (IA IE)^null^ (NSG-MHC-KO, stock no. 030547). All the procedures conducted on mice were approved by the Weill Cornell Medicine Institutional Animal Care and Use Committee and were consistent with the recommendations of the American Veterinary Medical Association and the National Institutes of Health Guide for the Care and Use of Laboratory Animals. All studies are reported in accordance with ARRIVE guidelines. To assess the specificity of PEN-221 to SSTR2 in vivo, we established a bilateral tumor model by subcutaneously implanting 1 × 10^6^ Jurkat-SSTR2 cells in the left flank and 1 × 10^6^ Jurkat-NT cells in the right flank. Thirty-five days after tumor inoculation, mice were treated with PEN-221 (25 µg in 100 µl water with 0.5% DMSO) every 3–4 days via tail vein or left untreated. Subcutaneous ATC tumor models were established by injecting 1 × 10^6^ FLuc-expressing 8505C tumor cells into the left flank. CAR T cells (10 × 10^6^ cells/mouse) were injected intravenously 5 days after the xenograft. PEN-221 (25 µg/mouse) were intravenously administered into CAR T-treated mice every 3–4 days between 20 and 33 days following T cell administration (5 injections total). Jurkat T and 8505C cells were prepared in 1:1 mixture of medium and Matrigel (Corning) for implantation, whereas primary human CAR T cells were injected via tail vein freshly after thawing. Tumor growth was monitored weekly via bioluminescence imaging using IVIS Spectrum in vivo imaging system (PerkinElmer). Images were acquired 10 min after intraperitoneal injection of 200 µl of 15 mg/ml D-luciferin (GoldBio) and analyzed using Living Image Software (PerkinElmer). Whole-body bioluminescence flux (photons/s) was used to estimate tumor burden. Tumors were measured weekly with a caliper and tumor volumes were estimated using the formula V = (length × width^2^)/2. Proliferation and biodistribution of CAR T cells were monitored weekly by PET/CT imaging using an Inveon micro-PET/CT scanner (Siemens Medical Solutions). The radiotracer ^18^F-NOTA-Octreotide (ABX Pharmaceuticals) was prepared as previously described^36^ and injected intravenously around 2 h before the imaging. A reference of 100 μL of 10%ID/cm^3^ was included for quantification. PET/CT images were processed with Amide v1.0.5 and Inveon Research Workplace software, including ROIs of the lungs and tumors to study CAR T cell expansion. Retro-orbital blood collection was performed weekly and serum cytokines were analyzed by a LEGENDplex™ kit (Biolegend) according to the manufacturer’s instructions. Animals reaching the humane endpoint (e.g., signs of illness or distress including ruffled fur, difficulty with diet, abnormal posture, or > 2000 mm^3^ tumor volume) were euthanized and all deaths recorded for survival analysis.

### Cytokine analysis

293T and 8505C cells (5 × 10^3^) were resuspended in 200 µl media in triplicate 96 wells and incubated at 37 °C in a 5% CO_2_ atmosphere for 24 h. Culture supernatants were collected and cytokine secretion was measured using a LEGENDplex™ kit (Biolegend). Cytokine concentrations were calculated using a standard curve (standards provided within the kit) following the manufacturers’ instructions.

## Statistics

Statistical analysis was performed using GraphPad Prism 9 software. An unpaired, two-tailed student’s t-test or a two-way ANOVA with Tukey’s multiple comparisons test was performed to evaluate differences between groups. Mouse survival was analyzed by a log-rank (Mantel-Cox) test. All data are represented as mean ± SD. *P* < 0.05 was considered statistically significant.

## Supplementary Information

Supplementary Information.

## Data Availability

All data generated or analyzed during this study are included in this published article.
