# Supplementary material for: SSTR2 as an anatomical imaging marker and a safety switch to monitor and manage CAR T cell toxicity
Source: Sci Rep. 2022 Dec 3;12:20932. doi: 10.1038/s41598-022-25224-z (PMC9719480; doi:10.1038/s41598-022-25224-z)
Supplement: Supplementary file 1 — Supplementary Information. [file 41598_2022_25224_MOESM1_ESM.docx]

**SSTR2 as an anatomical imaging marker and a safety switch to monitor and manage CAR T cell toxicity**

Yago Alcaina^1,3^, Yanping Yang^1,3^, Yogindra Vedvyas^1^, Jaclyn E. McCloskey^1^, Moonsoo M. Jin^1,2,*^

^1^Molecular Imaging Innovations Institute, Department of Radiology, Weill Cornell Medicine, New York, NY 10065, USA

^2^Department of Surgery, Weill Cornell Medicine, New York, NY 10065, USA

^3^These authors contributed equally: Yago Alcaina and Yanping Yang


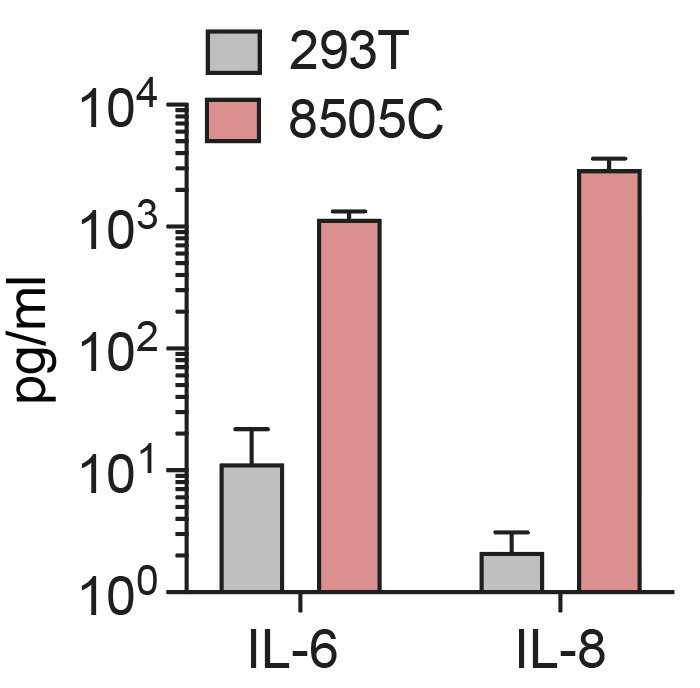


**Supplementary Figure 1.** 8505C cells release high concentration of IL-6 and IL-8 in culture supernantant. Cytokines were measured in supernatant of 293T and 8505C (5 × 10^3^ cells in 200 μL) culture at 24 hours. Data represent mean ± SD of 3–4 samples.
